# Participation in paediatric cancer studies: timing and approach to recruitment

**DOI:** 10.1186/1756-0500-6-191

**Published:** 2013-05-08

**Authors:** Tamika L Heiden, Helen D Bailey, Bruce K Armstrong, Elizabeth Milne

**Affiliations:** 1 Telethon Institute for Child Health Research, Centre for Child Health Research, The University of Western Australia, PO Box 855, West Perth WA 6872, Australia; 2 Sydney School of Public Health, The University of Sydney, Queen Elizabeth II Research Institute D02, The University of Sydney, Sydney, NSW 2006, Australia

**Keywords:** Recruitment, Pediatric cancer, Study invitation, Participation, Questionnaire

## Abstract

**Background:**

Participation in epidemiological studies has fallen significantly over the past 30 years; this has been attributed to a busier lifestyle and longer working hours. In case–control studies, participation among cases is usually higher than among controls due to the personal relevance. In Australia, between 2003 and 2011, we conducted three national population-based case–control studies of risk factors for childhood cancers; brain tumors, acute leukemia and neuroblastoma and Wilms’ tumor. In this sub-study, we aimed to investigate factors that may have influenced study participation and completeness of survey completion.

**Findings:**

The proportion of incident cases that were eligible to participate was lowest in the brain tumor study (Aus-CBT) (83.1%), as was the proportion of eligible families that consented (57%). The percentage of eligible cases that consented was highest in the leukemia study (Aus-ALL) (80.2%). The mode of invitation used was associated with families’ consent in each of the studies. Families invited in person, at clinic appointments, were more likely to consent than families invited by letter or phone. Timing of invitation following the child’s diagnosis differed among studies but, the likelihood of consent did not appear to be directly related to this. The return of questionnaires, completion of interview, and provision of DNA (blood sample) was highest in Aus-ALL (93%) and lowest in Aus-CBT (81%).

**Conclusions:**

Studies of childhood cancer, and possibly other childhood diseases, should arrange for the family to be invited in person and, where possible, by a doctor with whom they are familiar. Whilst telephone interviews are time consuming and costly, particularly for large studies, they should be preferred over questionnaires for obtaining complete data.

## Findings

### Background

Participation in epidemiological studies of all designs has fallen significantly over the past 30 years [1,2]. These falls in research participation have been attributed to longer working hours, more families with both parents working, and the demands of research on reduced family time [1]. In addition, increased telemarketing and greater numbers of study requests are believed to have created an “over-surveyed” society, in which people are less willing to volunteer their time [3-5].

In case–control studies, participation among cases is usually higher than among controls. This is probably due to the much greater personal relevance of the topic to cases [1]. Case participation is also falling, however; probably being affected by the factors affecting participation in general as well as specific factors, such as the growing frequency of opportunities and requests for people with particular illnesses and conditions to participate in research [1]. Studies involving children require the participation of parents and can have additional recruitment challenges. Parents of children with a chronic illness may view participation in epidemiological studies as risky because it is likely to evoke painful memories and feelings of guilt [6].

The way in which a person is approached to participate in a research study may influence their willingness to participate. Among methods used to invite participants to a study, “cold calls” or letters from unknown sources have been shown to be the least successful [7]. Face-to-face invitation has been highlighted as a preferred method of recruitment [1], and is more likely to be successful if it comes from someone known and trusted by the person being asked to participate [7].

Because it is important to maximize participation in order to draw valid inferences from research findings, we set out to investigate factors that may influence participation. To achieve this, we used information from three national Australian case–control studies of different childhood cancers conducted by our research group between 2003 and 2010. Here we describe the results of this novel sub-study.

### Methods

The Australian Study of Causes of Acute Lymphoblastic Leukaemia in Children (‘Aus-ALL’) [8] and The Australian Study of Childhood Brain Tumours (‘Aus-CBT’) were national population-based case–control studies, each conducted over a five year period. Aus-ALL cases were recruited between 2003 and 2007, and Aus-CBT cases between 2006 and 2010. ISET was an international pilot study of risk factors for Wilms’ tumour and neuroblastoma, initiated and coordinated by the International Agency for Research on Cancer (IARC) in Lyon, France and conducted in Australia in 2008.

Similar methods of recruitment were used in each study. Case families were identified through the ten paediatric oncology centres where virtually all children with malignancies in Australia are treated. In each study, collaborations were established with the designated lead clinician at each hospital before the grant application was submitted, and they estimated the numbers of diagnoses expected during the course of the study. Key issues and logistics relating to case recruitment were discussed and agreed with the lead clinicians and their clinical teams in face-to-face meetings or by telephone.

To be eligible to participate in any of the three studies, case families were required to reside in Australia, and to have at least one English-speaking biological parent, preferably the mother, available to complete the questionnaires; the child must be alive at the time of consent. In Aus-ALL, the child must also have achieved initial remission; remission occurred in more than 99% of notified cases.

The recruitment process used a series of electronic notification forms. The treating hospital notified the coordinating centre when i) a new case was diagnosed, ii) when the family was invited to participate (or the reason for not inviting them), and iii) when the family consented or declined to participate. Three modes of invitation were used to recruit families: a clinic visit (all three studies), an invitation letter from the treating clinician (Aus-CBT and ISET), or a telephone call from hospital staff (Aus-CBT), depending on the frequency and timing of clinic visits for the child’s cancer treatment and the preference of the treating hospital. Since children with ALL were seen by clinicians at weekly follow-up visits for a prolonged period, all families were invited at a clinic visit. Once parents consented to the study, the coordinating centre mailed out the study information and contacted families regarding data collection. In Aus-ALL and Aus-CBT, information on demographic variables and the exposures of interest were collected from parents in mailed, self-administered questionnaires and computer assisted telephone interviews. Blood samples for DNA analysis were collected from case children and their parents at the treating hospital. In ISET, similar information was collected by telephone interviews. DNA samples were not collected in the ISET pilot study because Aus-ALL and Aus-CBT had already shown that blood collection was feasible.

#### Ethics

Research and Ethics committees at all participating hospitals approved the studies. Parental consent, and the child’s consent when deemed appropriate, was obtained for the collection of data and biological samples.

#### Statistical analysis

In this study, we compared participation levels in three studies of different childhood cancers, and investigated the effects that invitation modes and timing had on consent and completeness of data collection. Where appropriate, logistic regression analyses and chi squared tests for differences in proportions were undertaken using PASW statistics v18 (SPSS Inc., IBM Corporation, Armonk, New York). Separate multivariable analyses were run with consent and questionnaire completion as dependent variables, and timing and mode of invitation as independent variables for both. Timing of invitation was treated as a categorical variable (<1 month, 1–3 months, 4–6 months, 7–18 months, or >18 months since diagnosis).

### Results

The numbers and percentages of notifications, eligible cases, invitations and consents for each of the studies are shown in Table 1. The proportion of incident cases who were eligible to participate was lowest for Aus-CBT, as was the proportion of eligible families who consented. The percentage of eligible cases who consented was highest in Aus-ALL.

**Table 1:** **Recruitment statistics in three Australian case**–**control studies of childhood cancer**

	**Aus**-**ALL**	**Aus**-**CBT**	**ISET**
Year(s) of diagnosis	2003-2006	2006-2010	2008
Type of cancer	ALL	Brain tumours	Wilms’ tumour or neuroblastoma
Number of cases ascertained in the study period	568	792	82
Eligible (% of notifications)	519 (91.4%)	658 (83.1%)	78 (95.1%)
Invitations (% of eligible)	484 (93.3%)	560 (85.1%)	70 (89.7%)
Consent (% of eligible)	416 (80.2%)	374 (57.0%)	52 (66.6%)

ALL –Australian Study of Acute Lymphoblastic Leukemia,

CBT – Australian Study of Childhood Brain Tumours,

ISET – International study of Embryonal Tumours.

The main reason for ineligibility in Aus-ALL was not having a parent who spoke English well enough to complete the questionnaires, while in Aus-CBT it was that the child died before their parents could be invited or give their consent to participate in the study, reflecting the aggressive nature of some types of CBT. There were very few ineligible families in ISET. Some eligible families were not invited because the treating doctor considered it inappropriate for medical or psychosocial reasons. This occurred less often in Aus-ALL (6.7% of eligible cases) than in Aus-CBT and ISET (14.9% and 10.3% respectively) (Table 1).

#### Factors influencing participation

In studies where more than one invitation mode was used (Aus-CBT and ISET), the mode of invitation was associated with consent. In both studies, families invited at clinic appointments were more likely to consent than families invited by letter or phone (Table 2). Furthermore, in Aus-CBT, a higher proportion of families invited by phone consented to participate (62%) than families invited by letter (48%); however, only 27 cases were invited by phone, so statistical testing was not done. Of the three studies, Aus-CBT had the lowest consent fraction among families invited in clinic.

**Table 2:** Number of families invited by each of the three invitation modes and number of families consenting based on invitation mode

	**Aus**-**ALL**	**Aus**-**CBT**	**ISET**
Clinic invitations (% of all invitations)	484 (100%)	363 (65%)	62 (89%)
Letter invitations (% of all invitations)		170 (30%)	8 (11%)
Phone invitations (% of all invitations)		27 (5%)	
Consent (% of all invited)	416 (86%)	374 (67%)	52 (74%)
Consent (% of invited in clinic)	416 (86%)	277 (76%)	49 (79%)
Consent (% of invited by letter)		81 (48%)	3 (38%)
Consent (% of invited by phone)		16 (62%)	

ALL –Australian Study of Acute Lymphoblastic Leukemia,

CBT – Australian Study of Childhood Brain Tumours,

ISET – International Study of Embryonal Tumours.

The time to invitation following the child’s diagnosis differed among studies. The median number of days between diagnosis and invitation in a clinic appointment was 84 days for Aus-ALL (~50 days from remission when cases became eligible), 89 days for Aus-CBT and 70 days for ISET. Few Aus-ALL invitations were made in the first month following diagnosis because of the required delay until after remission, but a greater proportion of Aus-ALL cases were invited within the first five months following diagnosis than in Aus-CBT and ISET (Table 3). The proportion of invitations in Aus-CBT and ISET was highest in the first month following diagnosis, but families continued to be invited over a long time.

**Table 3:** **Collection of data for Aus**-**ALL**, **Aus**-**CBT and ISET from participants invited in the clinic**

	**Aus**-**ALL**^**1**^	**Aus**-**CBT**^**1**^	**ISET**^**2**^
Number consented	416	374	52
Time lag between consent and data collection (median days)	58	50	25
% of cases invited within 5 months of diagnosis	93.6%	72.1%	73.7%
Data collected (% of consented)	388 (93%)	301 (81%)	43 (88%)
DNA Sample from child (% of consented)	415 (99%)	358 (96%)	
DNA sample from mother (% of consented)	414 (99%)	351 (94%)	
DNA samples from child and both parents (% of consented)	363 (87%)	278 (74%)	

^1 ^Data were collected in a self-administered postal questionnaire.

^2 ^Data were collected in a telephone interview.

In Aus-ALL, over 85% of cases invited in clinic within six months of diagnosis consented to the study. The corresponding proportions for Aus-CBT and ISET were 75% and 67% respectively. Generally, consent did not appear to be related to time of invitation within this six month interval. However, there was a suggestion that consent was less when Aus-CBT families were invited close to the first anniversary of the child’s diagnosis (65% in the three months around the anniversary) than in the first 11 months after diagnosis (75%; p = 0.30).

Logistic regression analysis of the associations of mode and time of invitation with consent in Aus-CBT cases showed that clinic invitations resulted in a greater proportion of consents than the other two modes of invitation combined, independently of the time since diagnosis. Families invited by telephone or letter were approximately 60 percent less likely to consent than those invited in clinic after adjusting for time since diagnosis (OR 0.39, 95% CI 0.25, 0.59). There was less evidence that time since diagnosis affected the likelihood of consent, after taking account of invitation mode (Table 4).

**Table 4:** Results obtained in logistic regression analysis for consent and questionnaire return

	**OR**	**95% CI**
**Consent**		
Mode of Invitation		
Clinic	1 (ref)	-
Letter or phone	0.39	0.25, 0.59
Timing of invitation*		
< 1 month since diagnosis	1 (ref)	-
3-6 months since diagnosis	0.56	0.28, 1.41
4-6 months since diagnosis	0.51	0.23, 1.12
7-18 months since diagnosis	0.50	0.24, 1.06
>18 months since diagnosis	0.40	0.16, 0.99
**Questionnaire Return**		
Mode of Invitation		-
Clinic	1 (ref)	
Letter	1.39	0.97, 1.99

OR = Odds Ratio, CI = Confidence Interval.

* Trend p-value with increasing time since diagnosis = 0.07.

#### Factors influencing completeness of participation

The completeness of participation – return of self-administered questionnaires, completion of interview and provision of DNA (blood sample) – also varied among the three studies (Table 3). In Aus-ALL, high proportions of families returned the questionnaires (93%) and provided the child’s DNA (99%) and DNA from both parents (87%). A high proportion of families (88%) completed the telephone interview in ISET. The proportion of families who provided survey data in Aus-CBT (81%) was less than in both Aus-ALL and ISET, and the proportion providing DNA for both parents (74%) was less than in Aus-ALL.

While the proportion of families who consented to Aus-CBT was highest in those invited in clinic (76%) and lowest in those invited by letter (48%, p <0.001) (Table 2), the proportion of consenting families who returned the questionnaires was somewhat higher among those who consented after being invited by letter (87.7%); than in those invited in clinic (78.3%) (p = 0.07), OR 1.39 (95% CI 0.97, 1.99) (Table 4).

Collection of data from parents was completed much sooner when done by telephone interviews, as in ISET, with the median time to study completion being only half the time taken for the return of the questionnaires in Aus-ALL and Aus-CBT (Table 3).

### Discussion

We compared the proportions of families who participated in three Australian case–control studies of childhood cancers between 2003 and 2010. Our results suggest that the nature and prognosis of the particular cancer, and its impact on the family, may influence the likelihood of being invited to take part by the doctor, and the decision to consent among those who are invited.

The length of time between diagnosis and invitation to participate varied by the type of cancer, and appeared to be related to the mode of invitation; in turn, the mode of invitation appeared to be the most important factor in the proportion of consenting cases. In all three studies, clinic invitations were most successful in recruiting case families, even after taking account of time since diagnosis. However, when comparing only those invited in clinic across the three studies, Aus-CBT had the lowest proportion of consents and Aus-ALL the highest. The differences in consent across the three studies were consistent with differences in the treatment regimens and prognosis, suggesting that participation in childhood cancer studies may be lower when the child’s prognosis is less favourable. Among ALL cases, 99% achieved remission, and the overall five year survival rate is high (80.6%) [9]. The five-year survival for Wilms’ tumour is approximately 89%, while for neuroblastoma it is 68% [9]. The treatment for CBT is often invasive and the outcome less favorable, with a 50-78% five-year survival [9]. In addition, children who do survive often have substantial long term morbidity. Therefore, when the expected course of the child’s cancer is unpredictable, parents may be less likely to participate in epidemiological research.

Invitations by letter were less successful than clinic invitations in obtaining consent. Previous epidemiological studies have also found face-to-face invitations to be most effective in recruitment [1]. Face-to-face invitations were not always possible in Aus-CBT and ISET due to the clinical course of the child’s disease, the treatment approach, and the frequency of clinic visits. The standard treatment protocols used in Aus-ALL, where case families had regular clinic visits for two years, provided more opportunities for invitation during a clinic visit. Invitation by a clinician during a clinic visit provided the opportunity to highlight the importance of the study to parents, thereby increasing the likelihood of consent [1]. Wherever possible, studies of childhood cancer, and possibly other childhood diseases, should arrange for the family to be invited in person and, where possible, by a doctor with whom they are familiar.

Completeness of data collection also differed across the three studies, and was highest in Aus-ALL. This has been observed in previous studies, with over 86% of participants providing data in a previous study of ALL [10]. Observations by project staff and feedback from participating families suggested that differences in prognosis and the degree to which families were coping influenced survey completion. Parents in Aus-CBT reported that they wanted to participate and therefore consented to the study, but later become overwhelmed and unable to complete the surveys. Completeness of participation in ISET was advantaged through the use of telephone interviews for data collection, a method which has been shown to result in better provision of data than self-administered questionnaires [11,12]. Thus, while telephone interviews are time consuming, costly and not always feasible for large studies [13], they should be preferred over questionnaires for obtaining complete data.

Timing of the invitation itself did not appear to influence the likelihood of participation except, perhaps, when it was near the anniversary of the child’s diagnosis. Invitation timing is possibly one of the most sensitive issues in childhood cancer research. Invitation in Aus-ALL was dependent on the child first reaching remission, thereby having the advantage of the family being invited in person and at a more appropriate stage, in terms of disease prognosis. In Aus-CBT responses to early invitations varied, with some families responding positively, and others declining to participate. Inviting families a long time after diagnosis, by any invitation mode, provided mixed results: some families said they were happy to help because their child was doing well, while other families did not want to revisit the painful memories associated with the diagnosis (unpublished observations). We suggest that the timing of invitation should be planned with a view to maximising the opportunity for in-person invitation, which, from our experience, will usually be soon after diagnosis.

In Aus-CBT, families consenting to research when invited by letter were more likely to complete the study questionnaires. Although an invitation by letter did not appear to be the best method of obtaining consent, it was valuable when the family needed more time to deal with the diagnosis, were no longer having regular clinic appointments or had moved to another treatment facility. It appeared that, if families had had this time and still decided to take part, they were more likely to return questionnaires than those who responded to an approach sooner after the diagnosis. Anecdotally, these families considered themselves lucky, most likely because treatment was out of the way and time had allowed them to come to terms with the diagnosis. They reported wanting to help prevent other families going through a similar experience (unpublished observations).

#### Limitations

This study has some limitations. No information, other than age and sex, was available for cases whose parents declined to participate. It is possible that factors such as socio-economic status may have influenced participation, but we were not able to collect this information. Similarly, it was not possible to survey parents about their reasons for declining to participate, and this could have added useful information. Our studies were conducted in different time periods across a span of seven years, and it is possible that falls in volunteerism in recent years, along with the increasing opportunities for families to participate in other studies and clinical trials, may have contributed to the lower participation in Aus-CBT, the most recent of our studies.

### Conclusions

Our results suggest that participation fractions in studies of childhood cancer vary by diagnosis. Further, among the variables studied, the mode of invitation was most likely to influence study participation. Understanding the contributions of these and other factors is likely to assist in increasing participation of case families in research studies, and thus increase the validity of the findings.

## Abbreviations

Aus-ALL: Australian Study of Acute Lymphoblastic Leukemia; Aus-CBT: Australian Study of Childhood Brain Tumours; ISET: International study of Embryonal Tumours; DNA: Deoxyribonucleic acid.

## Competing interests

The author(s) declare that they have no competing interests.

## Authors’ contributions

TH was involved in the acquisition, analysis and interpretation of data and drafted the manuscript. HB was involved in the acquisition and analysis of data. BA participated in the design of the study and helped with the manuscript drafting. LM participated in the study design and coordination, and helped to draft the manuscript. All authors read and approved the final manuscript.
